# Structural equation modeling as a tool to investigate correlates of extra-pair paternity in birds

**DOI:** 10.1371/journal.pone.0193365

**Published:** 2018-02-23

**Authors:** Nicholas M. A. Crouch, Roberta J. Mason-Gamer

**Affiliations:** 1 Dept. of Biological Sciences, University of Illinois at Chicago, 840 West Taylor St., MC066, Chicago, IL 60607, United States of America; 2 Department of Zoology, The Field Museum, 1400 S. Lake Shore Drive, Chicago, IL 60605, United States of America; University of South Carolina, UNITED STATES

## Abstract

Identifying relationships between variables in ecological systems is challenging due to the large number of interacting factors. One system studied in detail is avian reproduction, where molecular analyses have revealed dramatic variation in rates of extra-pair paternity—the frequency with which broods contain individuals sired by different males. Despite the attention the topic has received, identification of ecological predictors of the observed variation remains elusive. In this study we evaluate how structural equation modeling—which allows for simultaneous estimation of covariation between all variables in a model—can help identify significant relationships between ecological variables and extra-pair paternity. We estimated the correlation of eight different variables using data from 36 species of passerines by including them in six different models of varying complexity. We recover strong support for species with lower rates of male care having higher rates of extra-pair paternity. Our results also suggest that testes size, range size, and longevity all potentially have a relationship with rates of extra-pair paternity; however, interpretation of this result is more challenging. More generally, these results demonstrate the utility of applying structural equation modeling to understanding correlations among interacting variables in complex biological systems.

## Introduction

Variation in species mating systems can influence a range of ecological and evolutionary processes, including: strength of sexual selection, population demographics and variation in species traits [1–6]. Identifying ecological predictors of why mating systems in some taxa are so variable is therefore a major area of research. The application of molecular techniques to avian mating systems has revealed that socially-monogamous species, with a male and female paired at a nesting site, are infrequently genetically monogamous—chicks from a single brood are frequently sired by multiple males [7–10]. Numerous hypotheses have been generated to explain how extra-pair copulations may improve the fitness of individuals [11–14], but despite the considerable research, identification of ecological predictors of the observed variation remains challenging [9, 10].

Rates of extra-pair paternity (EPP) vary dramatically between avian species. There are some species where EPP is perhaps non-existent, for example in Carolina wrens (*Thryothorus ludovicianus* [15]). However, it appears uncommon for species to show no evidence of EPP, with most species showing low-levels of EPP [16]. Some species show extremely high rates of EPP, with most broods fathered by more than one male. For example, approximately three-quarters of broods of the Superb fairy-wren (*Malurus cyaneus*) are associated with multiple males [17]. This variation is not ubiquitously between distantly related taxa, members of the same genus can show similarly disparate rates of EPP [9, 16].

Numerous abiotic and biotic factors have been suggested to explain interspecific variation in EPP (reviews by [8, 9, 18]). These are wide ranging and include, but are not limited to, song performance [19], parental care [20], male brightness [21], and clutch size [22]. Despite the number of potential explanatory variables there is no clear consensus as to whether one can uniformly explain avian EPP. This is partly due to the ability of closely related taxa being similar in explanatory variables, but differing in their rates of EPP. For example, the blue tit (*Parus caeruleus*) and coal tit (*Parus ater*) have comparable testes size [23], suggested to influence rates of EPP [24], yet the rates of EPP in the coal tit are over double that seen in the blue tit [8].

Variation in life history between closely related taxa is not the only reason why identifying correlates of EPP is problematic. Additional challenges include, for example: a large number of explanatory factors [9]; methodological differences between studies; sampling bias in studied species [10]; and the potential for explanatory factors to covary, potentially leading to over-identification of explanatory factors [9]. Many studies that seek to identify ecological correlates of extra-pair paternity either focus on specific species, employ phylogenetic comparative methods, or apply meta-analytical techniques to try and parse out trends. Although these methods can give tremendous insight into statistically complex problems, there are still possible sources of error. For example, a potential limitation of multivariate statistical techniques is that if a model contains a series of confounding variables—multiple interactions between dependent variables—then potential signal between two traits of interest may be lost [25].

In this study we evaluate structural equation modeling (SEM) as a method to estimate whether eight important life history and morphological variables are correlated with EPP. SEM allows the specification of multiple predictive pathways between model variables to account for their influence on each other [26–28]. We derived six models representing different hypotheses about the relationships between variables, and compared their relative performance in explaining the data using a variety of model fitting techniques. We appraise the suitability of SEM for examining EPP by discussing the results of these analyses in the context of previous research.

## Materials and methods

We gathered data for 36 species of passerines (Passeriformes) from 15 families (Table 1) from a variety of sources. Levels of EPP are both higher and more variable in passerines compared to non-passerines [9]. We obtained EPP data, defined as the percentage of broods containing offspring sired by multiple males, from [29] and [18]. If a species was repeated between the two studies, we followed the more recent values [18]. We used percentage of broods to define EPP rather than percentage of young as these data were available for a larger number of taxa. These two approaches to defining EPP are highly correlated (Pearson r = 0.93, *n* = 19, data from [18]), and so the results are unlikely to be unaffected by which is used. We collected data on four potentially explanatory factors which have previously suggested to influence EPP: body size (grams [30]), longevity (years [31, 32]), male provisioning (percentage of broods fed by male [33]), and testes size (residual from regression between testes size and body size [23]). To these we added two potentially co-varying variables: range size (polygon size [34]) and altitude range (maximum—minimum values from across range [35]). Finally, we added two variables which are potentially sexually selected traits, therefore possibly involved in EPP, that are highly variable across the study taxa: range in clutch size (maximum—minimum clutch size [36] and song complexity.

**Table 1:** Study species.

Family	Species	Common Name	EPP
Acrocephalidae	*Acrocephalus arundinaceus*	Great reed warbler	6.00
	*Acrocephalus schoenobaenus*	Sedge Warbler	25.47
	*Cardinalis cardinalis*	Northern cardinal	13.51
	*Passerina cyanea*	Indigo bunting	70.00
Emberizidae	*Emberiza citrinella*	Yellowhammer	69.00
	*Emberiza schoeniclus*	Common reed bunting	64.00
	*Junco hyemalis*	Dark-eyed junco	28.34
	*Melospiza melodia*	Song sparrow	8.80
	*Passerculus sandwichensis*	Savannah sparrow	50.80
	*Spizella pusilla*	Field sparrow	15.10
	*Zonotrichia albicollis*	White-throated sparrow	12.80
	*Zonotrichia leucophrys*	White-crowned sparrow	31.00
Fringillidae	*Fringilla coelebs*	Common chaffinch	17.00
	*Haemorhous mexicanus*	House finch	14.30
Hirundidae	*Hirundo rustica*	Barn swallow	45.30
	*Progne subis*	Purple martin	27.50
Icteridae	*Agelaius phoeniceus*	Red-winged blackbird	55.00
	*Dolichonyx oryzivorus*	Bobolink	38.00
Mimidae	*Mimus polyglottos*	Northern mockingbird	8.00
Motacillidae	*Anthus spinoletta*	Water pipit	12.40
Muscicapidae	*Ficedula albicollis*	Collared flycatcher	38.95
	*Ficedula hypoleuca*	European pied flycatcher	14.50
	*Luscinia svecica*	Bluethroat	51.50
	*Oenanthe oenanthe*	Northern wheatear	29.00
Paridae	*Poecile atricapillus*	Black-capped chickadee	28.15
	*Cyanistes caeruleus*	Eurasian blue tit	49.18
	*Parus major*	Great tit	33.28
Parulidae	*Setophaga petechia*	Mangrove warbler	53.80
	*Setophaga ruticilla*	American redstart	59.00
	*Setophaga citrina*	Hooded warbler	35.30
Prunellidae	*Prunella modularis*	Dunnock	0.80
Sylviidae	*Phylloscopus sibilatrix*	Wood warbler	0.00
	*Phylloscopus trochilus*	Willow warbler	18.52
Troglodytidae	*Troglodytes aedon*	House wren	42.67
Turdidae	*Turdus merula*	Common blackbird	17.77
Vireonidae	*Vireo solitarius*	Blue-headed vireo	2.70

EPP is percentage of broods containing offspring sired by multiple males using data [18, 29].

We quantified a single metric for song complexity for each species before performing the SEM analyses in order to minimize model complexity given the low sample size of this study. Song complexity was defined using eight components of avian song, quantified from recordings downloaded from the online database xeno-canto (xeno-canto.org). Using the package *warbleR* [37] in the statistical program R [38] we measured: spectral entropy (complexity of the audio elements), spectral flatness (distribution of energy across spectral bands), modulation index (accumulated absolute difference between adjacent measurements of fundamental frequencies divided by the frequency range) and bandwidth (maximum—minimum frequency) of each recording. Additionally, we quantified song duration using the program Audacity [39] and the total number of notes, and number of unique notes, via visual inspection of recording sonograms from xeno-canto. Finally, we quantified trill rate by dividing the total number of notes produced by song length. We analyzed between 2 and 5 recordings for each species from disparate locations in their ranges. The recordings were not taken from the same location as the studies quantifying EPP for that species. We calculated the mean value for each of the eight elements of song complexity from all the recordings analyzed. Using the mean component values, we calculated a single overall metric for song complexity for each species as the sum of the score on each individual component, with each component scaled to be weighted equally.

We limited the analysis to eight potential explanatory factors, because including too many factors can potentially over-parameterize the models. These eight factors were chosen primarily on data availability, but they are also among those most frequently associated with differences in EPP [9, 10]. However, we did not limit included variables to those for which a significant relationship had been previously identified (for example, song complexity [29]), to test whether their inclusion in a path analysis would result in a identification of a significant relationship. Due to the expansive number of potential explanatory variables, those included here are not an exhaustive list, but provide a range of factors to test in the SEM framework. We did not include binary traits (e.g. song duetting, [40]), or variables that have additional confounding effects. For example, although species midpoint breeding latitude may correlate with EPP [41], the variation in latitude effects between hemispheres, and non-linear relationships between latitude and other factors (e.g. range size, [42]) could make interpretation of the results challenging at best [6]. SEM analyses can also be compromised if the causal variables are too highly correlated (multicollinearity [43]). We examined whether our analyses were susceptible to multicollinearity through a pairwise plot of the included variables S1 File).

We transformed our data to satisfy the requirements of SEM. Both range size and altitude range were log-transformed to obtain approximately normal distributions. To minimize differences in variances for the model components, we divided EPP rates and male feeding scores by 10. To correct for the statistical non-independence of species, the raw values for each factor were transformed by calculating phylogenetic independent contrasts (PIC [44]). Transformations were performed using phylogenetic data from [45] constructed using the backbone phylogeny of [46], implemented in the R package *ape* [47]. Both raw data with no correction for the relationships between species, and the transformed data were then passed to the SEM analysis [27]. We chose not to implement dedicated packages for calculating phylogenetic path analyses as they frequently estimate the λ parameter for calculating correlation structure. The λ parameter is notably problematic, and its inclusion would add another layer of uncertainty to this study.

### Statistical analysis

**Fig 1:**
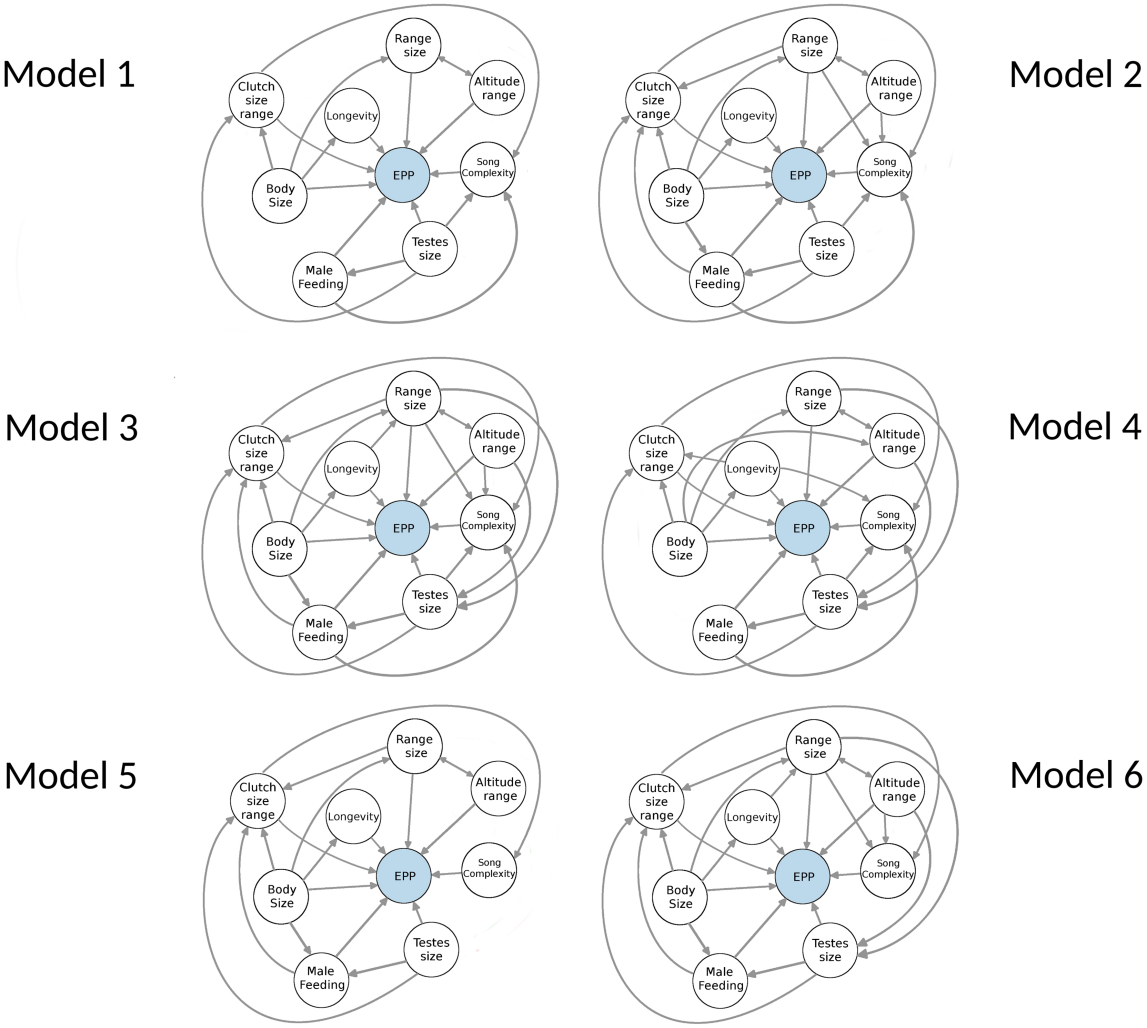
Graphical depictions of the six models tested in this study. All models include a connection between the six variables tested included here, but differed in the number of connections between variables. All models were fit with PIC and non-PIC transformed data.

The SEM framework allows for testing the contribution of a large number of variables while simultaneously accounting for potential correlations between them. Each unique combination of connections between variables constitutes a single model to fit to the data, with all models defined *a priori*. In this study, we defined six different models (Fig 1). Each included a direct link between the eight variables tested here (male feeding, testes size, body size, range in clutch size, longevity, range size, altitudinal range and song complexity) and rates of EPP. The models differed in the number of regressions between the eight explanatory variables. We chose connections between variables based primarily on previously identified relationships, for example between body size and longevity [48], but we did not specify whether any of these correlations were positive or negative *a priori*. None of the models included a link between body size and testes size as the testes size data from [23] were corrected for body size. We chose six models to evaluate the effect of network complexity on model fit and parameter estimation.

We solved each model using the R package *lavaan* [49] using maximum-likelihood estimation. To compare the relative fit of each model, we calculated AIC scores [50] to penalize the likelihood of each model by the respective number of parameters. We calculated three additional measures of fit for each model: *χ*^2^ (a general goodness-of-fit measure), root-mean square error of approximation (*rmsea*, which estimates the lack of fit between a tested model and the data given optimized parameters), and the comparative fit index (*cfi*, which compares the performance of each tested model to a ‘baseline’ model which assumes a zero correlation between all of the observed variables).

## Results

**Fig 2:**
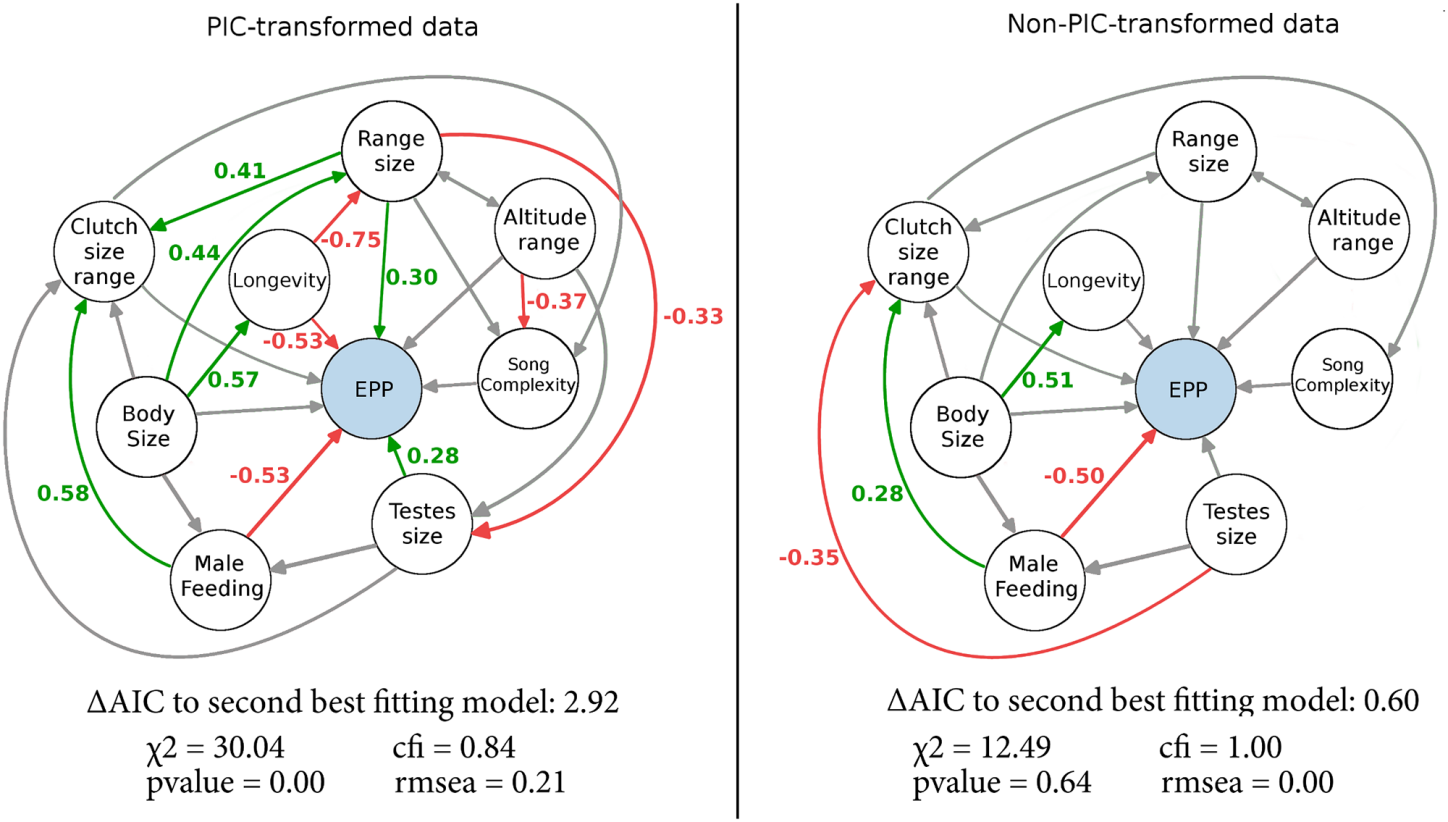
Graphical depiction of which of the six models were estimated to best explain the data when using PIC-transformed data (model 6, *left*) and non-PIC-transformed data (model 5, *right*). For clarity of display, the values for only those regressions estimated to be significant at the .05 level are shown. Regressions shown in gray are present in the model but not significant at the .05 level. All direct and indirect parameter estimates are provided in the supplementary material. Although the use of arrows in SEM figures suggests the directional effect of one variable on another, SEM analyses cannot identify cause and effect between variables.

The SEM analysis using PIC-transformed data recovered multiple significant relationships throughout the network (Fig 2, S1 File). Simpler models were generally favored, with models 3 and 6 estimated to be equally likely in explaining the data (Table 2). Despite a ΔAIC of 50.23 in estimated model fit, the parameter estimates were similar across all models. Testes size, range size, longevity, and male provisioning were all estimated to have large direct correlations with rates of EPP (p<0.05). Additionally, using only the best fitting model, two of the eight variables were estimated to have indirect correlations with rates of EPP. Body size had a negative indirect correlation with rates of EPP via longevity (Fig 2, regression weight −0.298). Longevity also had a similar negative indirect correlation with on rates of EPP via range size (estimated regression weight −0.227), despite not being estimated to be significant at the .05 level (p = 0.058). None of the remaining 51 estimated indirect correlations between the eight variables and EPP were significant at the .05 level, with the largest absolute regression weight being 0.13 (S1 File).

**Table 2:** Estimates of model fit, performance and parameters.

	Model	AIC	ΔAIC	*χ*^2^	df	*χ*^2^/df	pvalue	cfi	rmsea
*PIC*	6	200.07	0.00	30.04	12	2.50	0.00	0.84	0.21
	3	203.00	2.92	28.97	10	2.90	0.00	0.84	0.23
	5	218.89	18.82	54.86	15	3.66	0.00	0.66	0.28
	2	221.04	20.97	49.01	11	4.46	0.00	0.67	0.31
	4	236.23	36.16	72.20	15	4.81	0.00	0.51	0.33
	1	250.30	50.23	88.27	16	5.52	0.00	0.38	0.36
*No PIC*	5	1001.59	0.00	12.49	15	0.83	0.64	1.00	0.00
	1	1002.19	0.60	15.09	16	0.94	0.52	1.00	0.00
	2	1003.04	1.44	5.94	11	0.54	0.88	1.00	0.00
	6	1003.24	1.65	8.14	12	0.68	0.77	1.00	0.00
	3	1005.05	3.46	5.95	10	0.60	0.82	1.00	0.00
	4	1005.15	3.56	16.05	15	1.07	0.38	0.97	0.04
	Model	Body Size	Altitude Range	Testes Size	Range Size	Song Complexity	Clutch Size Range	Longevity	Male Feeding
*PIC*	6	0.003	−0.118	0.281*	0.280*	−0.108	0.113	−0.521*	−0.531*
	3	0.003	−0.126	0.299*	0.298*	−0.116	0.108	−0.554*	−0.564*
	5	0.003	−0.118	0.281*	0.300*	−0.109	0.105	−0.523*	−0.528*
	2	0.003	−0.115	0.274*	0.273*	−0.110	0.110	−0.508*	−0.518*
	4	0.003	−0.125	0.296*	0.295*	−0.112	0.107	−0.549*	−0.560*
	1	0.003	−0.119	0.283*	0.302*	−0.109	0.106	−0.526*	−0.532*
*No PIC*	5	−0.198	−0.180	0.237	0.219	−0.115	−0.042	−0.181	−0.475*
	1	−0.197	−0.188	0.247	0.228	−0.118	−0.044	−0.188	−0.496*
	2	−0.195	−0.187	0.246	0.227	−0.116	−0.044	−0.187	−0.491*
	6	−0.186	−0.179	0.234	0.216	−0.113	−0.043	−0.178	−0.469*
	3	−0.198	−0.189	0.248	0.229	−0.118	−0.044	−0.189	−0.497*
	4	−0.198	−0.190	0.249	0.230	−0.118	−0.044	−0.189	−0.498*

*Top*: Estimates of model fit for the six tested models sorted by ΔAIC, followed statistics for evaluating model performance. *df* is the degrees of freedom in each model, *cfi* is the comparative fit index, *rmsea* is the root-mean square approximation of error. In SEM, an insignificant pvalue for the chi-square test indicates good model performance. *Bottom*: standardized estimates of the direct correlations between the eight tested variables on EPP rates. Asterisks denote those parameters estimated to be significant at the 0.05 level.

In the SEM analysis using non-PIC-transformed data, there was little differentiation in the fit of the six models to the data, with all of the models covered by a ΔAIC of 3.56 (Table 2). As a result, each model could be considered equally likely in explaining the data. In similar fashion to the analysis using PIC-transformed data, each model had nearly identical parameter estimates, but in contrast to that analysis, only male provisioning was estimated to be significant at the .05 level (Table 2, S1 File). The estimated regression weights for testes size and range size were only slightly smaller than the PIC analysis, but the estimates for the correlation with longevity were considerably smaller (Table 2). None of the 19 indirect correlations between the eight variables on EPP from the best fitting model were estimated to be significant (S1 File). The largest absolute standardized indirect correlation was 0.11 (between body size and rates of EPP via male feeding). Transforming the data using PIC before using SEM had a dramatic effect on estimated model fit. The analysis of non-PIC-transformed data shows better performance fit in terms of all four measures (*χ*^2^, *pvalue*, *cfi* and *rmsea*). Nevertheless, the parameter estimates from the two sets of models are broadly comparable with the notable exception of longevity. Transforming the data increased the regression weight of longevity by 0.36 on average (ranging between 0.32 and 0.41, Table 2). The r^2^ values for the endogenous model variables for both transformed and transformed data are presented in Table 3.

**Table 3:** *r*^2^ values for the endogenous variables for PIC-transformed data (*top*) and non-PIC-transformed data (*bottom*).

	Model	EPP	Song Complexity	Clutch Size Range	Testes Size	Range Size	Male Feeding	Altitude Range	Longevity
*PIC*	6	0.724	0.152	0.604	0.096	0.383	0.068		0.321
	3	0.728	0.184	0.604	0.096	0.383	0.068	-	0.321
	5	0.700	0.002	0.585	-	0.000	0.083	-	0.321
	2	0.695	0.198	0.585	-	0.000	0.083	-	0.321
	4	0.743	0.251	0.354	-	0.000	0.065	0.112	0.321
	1	0.730	0.042	0.127	-	0.000	0.065	-	0.321
*No PIC*	5	0.426	0.004	0.269	-	0.000	0.056	-	0.263
	1	0.475	0.105	0.192	-	0.000	0.007	-	0.263
	2	0.430	0.169	0.269	-	0.000	0.056	-	0.263
	6	0.424	0.118	0.260	0.077	0.005	0.054	-	0.263
	3	0.438	0.166	0.260	0.077	0.005	0.054	-	0.263
	4	0.488	0.101	0.253	-	0.000	0.007	0.037	0.263

Variables do not have an *r*^2^ value if it was not on the left-hand side of a regression equation. This is depicted graphically as a variable not having an arrow pointing at it, see Fig 1.

## Discussion

Identifying interactions between variables in biological systems is challenging due to the number of potential explanatory factors and their ability to covary. In this study we used SEM to estimate the correlation between eight variables on rates of EPP while simultaneously estimating the extent to which they co-vary each other. When phylogenetic independent contrasts were performed prior to SEM analysis, testes size, range size, and species longevity were estimated to be significant predictors of rates of EPP. Although the estimates were similar for testes size and range size in the analysis where no PIC was performed, only male care was estimated to be a significant predictor of EPP. Both analyses showed a strong negative relationship between male care and rates of EPP.

An important consideration when interpreting the results of SEM analyses is that it is not possible to distinguish cause and effect. This is because there is no manipulation of an independent variable, and variables can be considered ‘independent’ and ‘dependent’ at the same time for different parts of the same model. Therefore, although SEM models are almost ubiquitously depicted with arrows, suggesting the directional influence of one variable on another, these only reflect *a priori* expectations about how variables may interact. Instead, SEM analyses fit parameters to the observed data to determine which variables of the model appear to be interacting.

Interpretation of the results from this study also requires consideration of two important methodological points: controlling for statistical non-independence of species before performing SEM, and how well each of the models are estimated to explain the data. The long-established idea that species do not represent statistically independent data points [44] means that statistical transformation to account for shared ancestry should be performed prior to the data being passed to the models [27]; however, it is not always performed [28]. If species traits are not evolving under Brownian Motion, then PIC transformation may not be the most appropriate method for transforming the data [51]. Although PIC allows the SEM models to account for shared ancestry among species, the fit estimates of models based on PIC transformed data were all poor, while non-PIC transformed data yielded better-fitting models. Thus, although PIC-transformation might be appropriate, the parameter estimates may not accurately describe the data. It is unclear why the appropriate data transformation resulted in such a pronounced drop in estimated model performance. Interpretation of the results must therefore incorporate consideration of both the data used and whether the parameter estimates appear to accurately describe the data.

Numerous studies have provided evidence that, in species where males provide less parental care, rates of EPP are higher [6, 16, 52–54]. In this study we also recover a strong negative relationship between male care and rates of EPP, with the estimated regression weight only marginally smaller when non-PIC transformed data are used. Greater parental care by males reduces the amount of time available to seek extra-pair copulations, with low EPP rates increasing the chance that males are raising their own young [55]. However, this hypothesis implies that, even though females may actively pursue extra-pair matings, rates of EPP are differentially controlled by male strategies. If instead males are responding to the strategies of females, then the amount of care provided by males could be in response to a perceived idea of how many chicks in a brood they have sired [56–58], even if feeding efforts increase when the female has mated with more than one male [59, 60]. Different studies have suggested that males (of the study species) cannot recognize, or at least do not discriminate against, unrelated chicks [61, 62], so we can’t determine which hypothesis best explains the observed relationship; the results simply provide strong evidence that rates of EPP are related to male care. One potential issue is that there may be bias—out of 18 species for which data were available, only two lacked any form of mate guarding (*Vireo solitarius* and *Agelaius phoenicus*, [52, 63, 64]). Mate guarding by males likely means a greater investment in their social brood, potentially reducing EPP. Thus, care must be taken in interpreting the results in case our data do not equally represent all possibilities of potentially confounding variables.

Our results suggest a positive relationship between testes size and rates of EPP (Fig 2), although the magnitude of the correlation differs between analyses. Only the analysis using PIC-transformed data recovers a significant regressions (p<0.05); however, the estimated regression weights between the two analyses differ by only 0.04 on average (Table 2). A comparison of the regression weights is important because, although *p*-value significance can be a useful yardstick for interpretation of results, there is a growing consensus that research should be moving away from the strict rigidity of only considering results significant at the 0.05 level [65, 66]. In this case, the inference of a positive relationship between testes size and rates of EPP does have biological merit. It could be driven by breeding synchrony, as the species in this study are predominantly temperate breeders which breed more synchronously [41]. A large number of males breeding at the same time increases the potential for sperm competition which can lead to an increase in testes size [9, 24]. This hypothesis is complicated by a lack of a definitive correlation between breeding synchrony and rates of EPP (reviewed by [9, 10]). Furthermore, temperate passerine species have larger testes than those from the tropics [41], so the prevalence of temperate species in this study may influence the relationship between EPP and testes size in an unknown manner.

As with testes size, the estimated positive relationship between EPP and range size is almost identical between the two analyses (Table 2), but biological interpretation of the relationship is more challenging. Increasing range size correlates with increasing local abundance [67, 68], so it could represent increasing breeding density. However, there is no strong evidence for a relationship between breeding density and rates of EPP [8, 69]. Furthermore, increasing breeding density should increase testes size [23], and our results suggest a negative relationship (Fig 2). One hypothesis, therefore, is that the greater dispersal ability of species with larger ranges [70] facilitates movement between nest sites and subsequently increases extra-pair copulations.

Determining whether species longevity is a significant predictor of EPP based on our results is somewhat equivocal as a large, a significant regression weight was only estimated in the analysis using PIC-transformed data. The data transformation is likely affecting longevity due to its strong correlation with body size (Fig 2), which has strong phylogenetic signal [71]. Correcting for the strong non-independence of body size could therefore affect the estimated correlation between longevity and EPP in turn. At the same time, the low estimated model fit for the models using PIC-transformed data means that the parameter estimates may not accurately describe the data. Nevertheless, there is also a theoretical basis for predicting that longer lived species should exhibit higher rates of EPP; males can benefit by investing less in a single brood if there is both a chance that some of the chicks were sired by another individual, and he has a chance to breed again in a subsequent year (reviewed by [10]). Our results are consistent with this idea, but this hypothesis relies on the male having knowledge about his level of paternity (discussed previously), and would mean that this is principally a male-driven strategy. Instead, if high rates of EPP were a species-level adaptation to reduced longevity then this may better incorporate female-based strategies to seeking extra-pair copulations.

Our application of SEM to these EPP data demonstrate its utility as a statistical tool for identifying ecological correlates; however, there is scope for improvement. First, we need to seek biological explanations for those trends that are currently unexplained—principally the relationship between species range size and rates of EPP. The second focus should be on increasing sample size, as analyses like SEM can be sensitive to low sample sizes [72]. We suspect this problem is further compounded in these analyses by the predominance of temperate species in the data (which reflects that the majority of research quantifying rates of EPP has been performed at European and North American research institutions).

Future research can also aim to incorporate intra-specific variation in EPP as it can vary tremendously [18]. For example, rates of EPP in the Reed bunting (*Emberiza schoeniclus*) can vary between 54% and 88% of broods in different populations [6]. Variation can be due to, for example: habitat [73], genetic similarity to partner [74], age of individuals [75], and breeding density [76]. Incorporating intraspecific variation into analyses as presented here is non-trivial as the other model variables may also vary between populations. Therefore, simply changing the EPP value used in the model would not be biologically meaningful. Perhaps the best approach for future research would be, where possible, to analyze data at the population level which could account for these potential differences.

Our results nevertheless demonstrate that SEM can be applied to highly complicated biological networks through identification of novel (range size) and established (male provisioning) correlates of EPP, while accounting for covariation between variables (e.g. body size and longevity, [48]). Furthermore, as SEM considers multiple variables simultaneously, the relative influence of these different variables can be estimated. These characteristics of SEM mean it has the potential to address questions on a range of topics, including: carbon cycling [77], relationships between organismal traits [78], and predator-prey interactions [79]. It is with increasing data availability, however, that the widespread utility of SEM will undoubtedly increase.

## Supporting information

S1 File Supplementary tables. (PDF)

S2 File A markdown file showing how the analyses were performed. (PDF)

S3 File An R file containing the specification of the six models plus an additional function used in the analyses. (R)

S4 File Data used in this study. (CSV)
