# Supplementary material for: Structural equation modeling as a tool to investigate correlates of extra-pair paternity in birds
Source: PLoS One. 2018 Feb 23;13(2):e0193365. doi: 10.1371/journal.pone.0193365 (PMC5825100; doi:10.1371/journal.pone.0193365)
Supplement: S1 File — (PDF) [file pone.0193365.s001.pdf]

Structural equation modeling as a tool to investigate correlates of extra-pair paternity in birds - Supplementary Material

Supplementary Figures

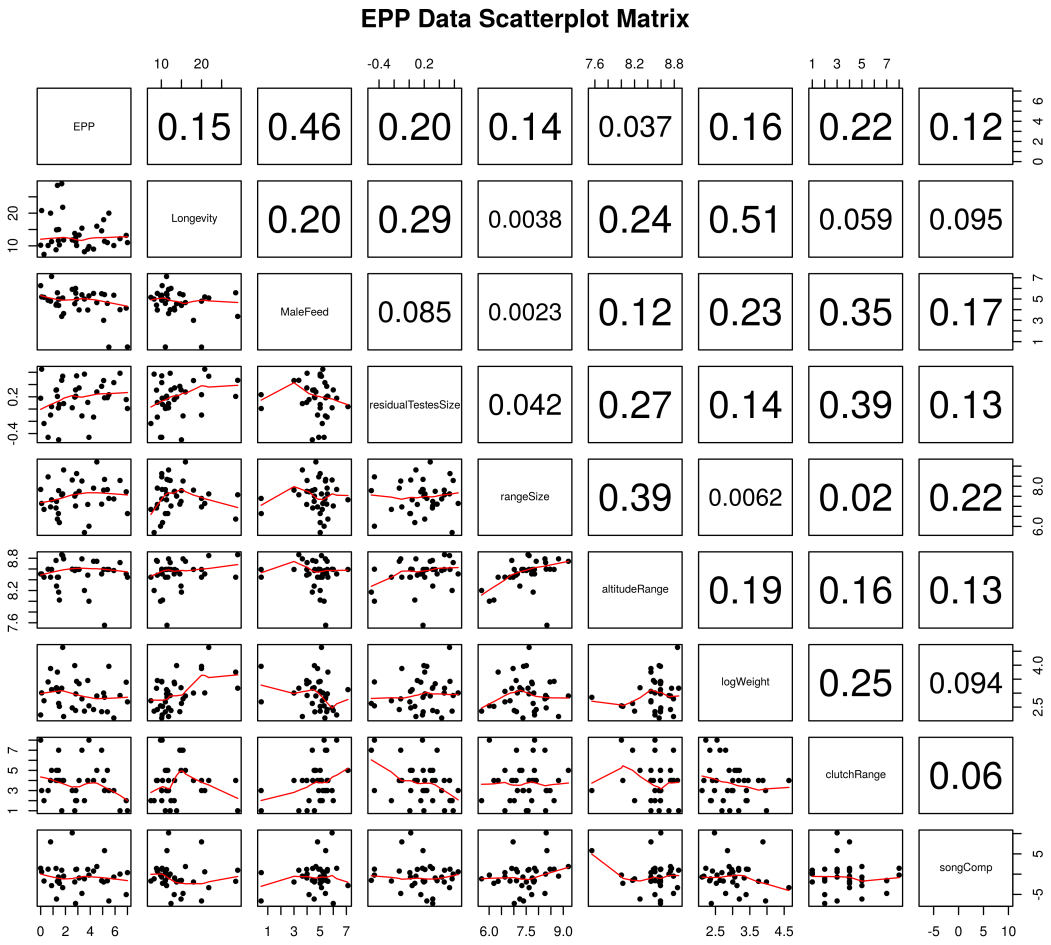

Figure S1: Pairwise correlations between the nine variables included in this study.

lhs	rhs	est.std	se	z	pvalue
EPPrate	rangeClutchSize	0.11	0.14	0.74	0.46
EPPrate	songComplexity	-0.11	0.10	-1.13	0.26
EPPrate	testesSize	0.28	0.10	2.84	0.00
EPPrate	bodySize	0.00	0.12	0.02	0.98
EPPrate	altitude_range	-0.12	0.12	-1.01	0.31
EPPrate	range_size	0.30	0.15	2.00	0.05
EPPrate	male_feeding	-0.53	0.13	-4.14	0.00
EPPrate	longevity	-0.53	0.15	-3.60	0.00
songComplexity	rangeClutchSize	-0.25	0.17	-1.43	0.15
songComplexity	range_size	0.32	0.18	1.74	0.08
songComplexity	altitude_range	-0.37	0.16	-2.31	0.02
rangeClutchSize	testesSize	-0.10	0.11	-0.87	0.39
rangeClutchSize	bodySize	-0.08	0.11	-0.71	0.48
rangeClutchSize	male_feeding	0.58	0.10	5.93	0.00
rangeClutchSize	range_size	0.41	0.11	3.77	0.00
range_size	bodySize	0.43	0.15	2.82	0.00
range_size	longevity	-0.75	0.12	-6.05	0.00
longevity	bodySize	0.57	0.11	4.93	0.00
male_feeding	testesSize	-0.21	0.16	-1.30	0.19
male_feeding	bodySize	-0.14	0.16	-0.89	0.38
testesSize	range_size	-0.33	0.17	-1.97	0.05
testesSize	altitude_range	0.20	0.17	1.17	0.24

Table S1: All direct effects for model 6 using PIC transformed data. ‘lhs’ and ’rhs’ correspond to the different sides of the regression equation, ‘est.std’ is the standardized parameter estimate, ‘se’ is the standard error of the parameter, and ‘z’ is the estimated parameter value divided by the standard error.

path	est.std	se	z	pvalue
maleFeeding → rangeClutch → songComplexity → EPP	-0.02	0.02	-0.64	0.52
maleFeeding → rangeClutch → EPP	0.06	0.08	0.73	0.47
rangeSize → songComplexity → EPP	-0.04	0.04	-0.93	0.35
rangeSize → clutchRange → EPP	0.04	0.06	0.72	0.47
rangeSize → clutchRange → songComplexity → EPP	0.01	0.01	0.85	0.39
rangeSize → testesSize → EPP	-0.09	0.06	-1.52	0.13
rangeSize → testesSize → maleFeeding → rangeClutch → EPP	0.00	0.01	0.60	0.55
rangeSize → testesSize → maleFeeding → rangeClutch → songComplexity_EPP	0.00	0.00	0.67	0.50
rangeSize → testesSize → maleFeeding → EPP	-0.04	0.04	-1.02	0.31
rangeSize → testesSize → rangeClutch → EPP	0.00	0.01	0.53	0.59
rangeSize → testesSize → rangeClutch → songComplexity_EPP	0.00	0.00	0.58	0.56
clutchRange → songComplexity → EPP	-0.03	0.04	-0.65	0.52
altitudeRange → songComplexity → EPP	0.04	0.04	1.00	0.32
altitudeRange → testesSize → EPP	0.06	0.05	1.05	0.29
altitudeRange → testesSize → maleFeeding → rangeClutch → EPP	-0.00	0.00	-0.55	0.58
altitudeRange → testesSize → maleFeeding → rangeClutch → songComplexity → EPP	-0.00	0.00	-0.61	0.54
altitudeRange → testesSize → maleFeeding → EPP	0.02	0.03	0.84	0.40
altitudeRange → testesSize → rangeClutch → EPP	-0.00	0.00	-0.50	0.62
altitudeRange → testesSize → rangeClutch → songComplexity → EPP	-0.00	0.00	-0.54	0.59
bodySize → longevity → EPP	-0.30	0.11	-2.72	0.01
bodySize → longevity → rangeSize → EPP	-0.13	0.07	-1.73	0.08
bodySize → longevity → rangeSize → songComplexity → EPP	0.01	0.02	0.90	0.37
bodySize → longevity → rangeSize → rangeClutch → EPP	-0.02	0.03	-0.71	0.48
bodySize → longevity → rangeSize → rangeClutch → songComplexity → EPP	0.01	0.01	0.87	0.38
bodySize → longevity → rangeSize → testesSize → EPP	0.04	0.03	1.39	0.17
bodySize → longevity → rangeSize → testesSize → maleFeeding → EPP	0.02	0.02	0.98	0.33
bodySize → longevity → rangeSize → testesSize → maleFeeding → rangeClutch → EPP	-0.00	0.00	-0.59	0.56
bodySize → longevity → rangeSize → testesSize → maleFeeding → rangeClutch → songComplexity → EPP	-0.00	0.00	-0.65	0.51
bodySize → longevity → rangeSize → testesSize → rangeClutch → EPP	-0.00	0.00	-0.53	0.60
bodySize → longevity → rangeSize → testesSize → rangeClutch → songComplexity → EPP	-0.00	0.00	-0.57	0.57
bodySize → rangeClutch → EPP	-0.01	0.02	-0.51	0.61
bodySize → rangeClutch → songComplexity → EPP	-0.00	0.00	-0.55	0.58
bodySize → rangeSize → clutchRange → EPP	0.02	0.03	0.70	0.48
bodySize → rangeSize → clutchRange → songComplexity → EPP	0.00	0.01	0.82	0.41
bodySize → rangeSize → EPP	0.13	0.08	1.62	0.11
bodySize → maleFeeding → EPP	0.08	0.09	0.84	0.40
bodySize → maleFeeding → rangeClutch → EPP	-0.01	0.02	-0.56	0.58
bodySize → maleFeeding → rangeClutch → songComplexity → EPP	-0.00	0.00	-0.61	0.54
testesSize → clutchRange → songComplexity → EPP	-0.00	0.00	-0.61	0.54
testesSize → clutchRange → EPP	-0.01	0.02	-0.56	0.58
testesSize → maleFeeding → EPP	0.11	0.09	1.24	0.22
testesSize → maleFeeding → clutchRange → EPP	-0.01	0.02	-0.64	0.53
testesSize → maleFeeding → clutchRange → songComplexity → EPP	-0.00	0.00	-0.72	0.47
longevity → rangeSize → EPP	-0.23	0.12	-1.90	0.06
longevity → rangeSize → songComplexity → EPP	0.03	0.03	0.92	0.36
longevity → rangeSize → rangeClutch → EPP	-0.03	0.04	-0.72	0.47
longevity → rangeSize → rangeClutch → songComplexity → EPP	0.01	0.01	0.89	0.37
longevity → rangeSize → testesSize → EPP	0.07	0.05	1.47	0.14
longevity → rangeSize → testesSize → maleFeeding → EPP	0.03	0.03	1.00	0.32
longevity → rangeSize → testesSize → maleFeeding → rangeClutch → EPP	-0.00	0.01	-0.60	0.55
longevity → rangeSize → testesSize → maleFeeding → rangeClutch → songComplexity → EPP	-0.00	0.00	-0.66	0.51
longevity → rangeSize → testesSize → rangeClutch → EPP	-0.00	0.00	-0.53	0.59
longevity → rangeSize → testesSize → rangeClutch → songComplexity → EPP	-0.00	0.00	-0.58	0.56

Table S2: All indirect effects for model 6 using PIC transformed data. 'est.std' is the standardized parameter estimate, 'se' is the standard error of the parameter, and 'z' is the estimated parameter value divided by the standard error.

lhs	rhs	est.std	se	z	pvalue
EPPrate	rangeClutchSize	-0.04	0.15	-0.30	0.76
EPPrate	songComplexity	-0.12	0.13	-0.93	0.35
EPPrate	testesSize	0.25	0.14	1.77	0.08
EPPrate	bodySize	-0.20	0.15	-1.29	0.20
EPPrate	altitude_range	-0.19	0.14	-1.31	0.19
EPPrate	range_size	0.23	0.14	1.68	0.09
EPPrate	male_feeding	-0.50	0.13	-3.93	0.00
EPPrate	longevity	-0.19	0.15	-1.29	0.20
songComplexity	rangeClutchSize	-0.06	0.17	-0.36	0.72
rangeClutchSize	testesSize	-0.35	0.14	-2.54	0.01
rangeClutchSize	bodySize	-0.14	0.15	-0.94	0.35
rangeClutchSize	male_feeding	0.28	0.14	2.00	0.05
rangeClutchSize	range_size	-0.00	0.14	-0.03	0.97
range_size	bodySize	0.01	0.17	0.04	0.97
longevity	bodySize	0.51	0.12	4.17	0.00
male_feeding	testesSize	-0.05	0.16	-0.34	0.74
male_feeding	bodySize	-0.22	0.16	-1.39	0.16

Table S3: All direct effects for model 5 using untransformed data. 'lhs' and 'rhs' correspond to the different sides of the regression equation, 'est.std' is the standardized parameter estimate, 'se' is the standard error of the parameter, and 'z' is the estimated parameter value divided by the standard error.

Path	est.std	se	z	pvalue
bodySize → longevity → EPP	-0.10	0.08	-1.22	0.22
bodySize → rangeSize → EPP	0.00	0.04	0.04	0.97
bodySize → rangeSize → clutchRange → EPP	0.00	0.00	0.03	0.98
bodySize → rangeSize → clutchRange → songComplexity → EPP	-0.00	0.00	-0.03	0.98
bodySize → rangeClutch_ → songComplexity → EPP	-0.00	0.00	-0.31	0.75
bodySize → rangeClutch → EPP	0.01	0.02	0.29	0.78
bodySize → maleFeeding → EPP	0.11	0.09	1.25	0.21
bodySize → maleFeeding → rangeClutch → EPP	0.00	0.01	0.29	0.77
bodySize → maleFeeding → rangeClutch → songComplexity → EPP	-0.00	0.00	-0.32	0.75
testesSize → maleFeeding → EPP	0.03	0.08	0.34	0.74
testesSize → maleFeeding → rangeClutch → EPP	0.00	0.00	0.22	0.82
testesSize → maleFeeding → rangeClutch → songComplexity → EPP	-0.00	0.00	-0.24	0.81
testesSize → clutchRange → EPP	0.02	0.05	0.30	0.77
testesSize → clutchRange → songComplexity → EPP	-0.00	0.01	-0.33	0.74
maleFeeding → rangeClutch → EPP	-0.01	0.04	-0.30	0.77
maleFeeding → clutchRange → songComplexity → EPP	0.00	0.01	0.33	0.74
clutchRange → songComplexity → EPP	0.01	0.02	0.33	0.74
rangeSize → clutchRange → EPP	0.00	0.01	0.03	0.97
rangeSize → clutchRange → songComplexity → EPP	-0.00	0.00	-0.03	0.97

Table S4: All indirect effects for model 5 using untransformed data. 'est.std' is the standardized parameter estimate, 'se' is the standard error of the parameter, and 'z' is the estimated parameter value divided by the standard error.
