# Supplementary material for: Structural equation modeling as a tool to investigate correlates of extra-pair paternity in birds
Source: PLoS One. 2018 Feb 23;13(2):e0193365. doi: 10.1371/journal.pone.0193365 (PMC5825100; doi:10.1371/journal.pone.0193365)
Supplement: S2 File — (PDF) [file pone.0193365.s002.pdf]

Structural equation modeling as a tool to investigate correlates of extra-pair paternity in birds

Contents

Data preparation **2**

Model fitting **4**

Model fitting results **5**

Parameters of best fitting model **7**

Model fitting with PIC data **11**

References **14**

Data preparation

First, the required libraries and data need to be loaded

```
# Load library
library(lavaan)
library(semPlot)
library(qgraph)

# Load the data
dat <- read.csv("sem_epp_data.csv")

# Define range in clutch size
dat$clutchRange <- dat$MaxClutch - dat$MinClutch
```

In order to minimize the complexity of the model we will calculate song complexity before performing the SEM analyses. The score a species receives for song complexity is the sum of its scaled score for each of the eight component parts. Therefore, for a species to receive a high score, it must have a high score across all aspects of song complexity quantified here.

```
# Extract just the eight components of song complexity used here
songElements <- dat[,c("overallSongDuration", "numberElements", "maxNumberTrills",
             "sp.ent", "sfm","modindx", "bandwidth", "trillRate")]

# The reverse of sfm (spectral flatness) is used, as higher untransformed values
# correspond to songs which are more similar across all bands
songElements$sfm <- 1 - songElements$sfm

# Scale each of the elements of song complexity
songElements <- apply(songElements, 2, scale)

# Calculate the row sums for the scaled data frame
songComp <- rowSums(songElements)
# Note: It makes no difference if all the values are made positive
# before calculating the rowsums

# Add back into the original data frame
dat$songComp <- songComp
```

Some of the rows in the data fame contain missing data (almost exclusively male feeding rates), these rows will now be removed

```
dat <- dat[complete.cases(dat),]

nrow(dat) # leaves 36 species
```

```
## [1] 36
```

SEM models require that the variances of each of the component elements are similar, and assumes that the data are normally distributed. In order to satisfy these critera, the following transformations are performed:

```
dat$rangeSize <- log(dat$rangeSize)

dat$altitudeRange <- log(dat$altitudeRange)

dat$EPP <- dat$EPP / 10
```

```
dat$MaleFeed <- dat$MaleFeed / 10
```

Next the phylogenetic data will be loaded. As we removed some taxa from our analyses, those taxa will have to be pruned from the phylogeny

```
library(ape)

phy <- read.nexus("sampledPhylo.nex")

dat$Species <- gsub(" ","_",dat$Species)

v <- phy$tip.label %in% dat$Species

dropSpp <- phy$tip.label[v==FALSE]

phy <- drop.tip(phy, dropSpp)

plot(phy)
```

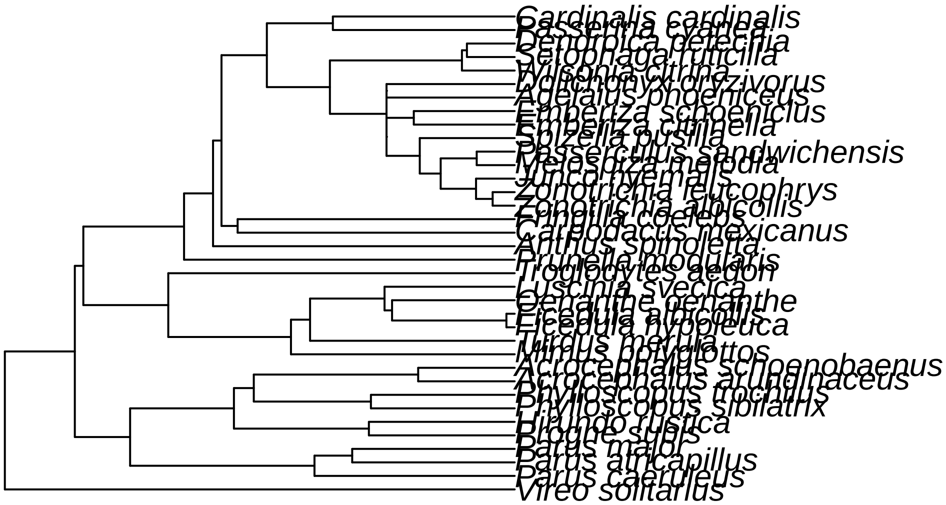

We can now perform PIC on each of the component parts of our model

```
PICdat <- dat
PICdat <- PICdat[-1,]# Perfoming PIC creates n-1 vaues

PICdat$rangeSize <- pic(dat$rangeSize, phy)
PICdat$logWeight <- pic(dat$logWeight, phy)
PICdat$residualTestesSize <- pic(dat$residualTestesSize, phy)
PICdat$altitudeRange <- pic(dat$altitudeRange, phy)
PICdat$clutchRange <- pic(dat$clutchRange, phy)
PICdat$EPP <- pic(dat$EPP, phy)
PICdat$songComp <- pic(dat$songComp, phy)
PICdat$MaleFeed <- pic(dat$MaleFeed, phy)
PICdat$Longevity <- pic(dat$Longevity, phy)
```

Model fitting

All of the SEM models employed in this study have been saved in a separate file which will be read in here. Note: in no models is there a link between body size and testes size as the testes size data from Pitcher, Dunn, and Whittingham (2005) are scaled by body size. Graphs of all the models tested are provided in a separate pdf in the supplementary material.

```
source("SEMmodels.R")
```

The models vary in the number of connections between the varibles within the model. The fit of each model to the data can now be tested.

```
model.1.fit <- lavaan::sem(model1, data=dat)
model.2.fit <- lavaan::sem(model2, data=dat)
model.3.fit <- lavaan::sem(model3, data=dat)
model.4.fit <- lavaan::sem(model4, data=dat)
model.5.fit <- lavaan::sem(model5, data=dat)
model.6.fit <- lavaan::sem(model6, data=dat)
```

Have all of the models converged on a solution?

```
model.1.fit@optim$converged
```

```
## [1] TRUE
```

```
model.2.fit@optim$converged
```

```
## [1] TRUE
```

```
model.3.fit@optim$converged
```

```
## [1] TRUE
```

```
model.4.fit@optim$converged
```

```
## [1] TRUE
```

```
model.5.fit@optim$converged
```

```
## [1] TRUE
```

```
model.6.fit@optim$converged
```

```
## [1] TRUE
```

Model fitting results

Create a data frame to store the data on how well each of the models explain the data.

```r
model.fit.results <- as.data.frame(matrix(NA, ncol=8, nrow=6))
colnames(model.fit.results) <- c("Model", "AIC", "deltaAIC", "chisq", "df", "pvalue", "cfi", "rmsea")

# AIC scores
model.fit.results$Model <- seq(1, 6, 1)
model.fit.results[1,2] <- AIC(model.1.fit)
model.fit.results[2,2] <- AIC(model.2.fit)
model.fit.results[3,2] <- AIC(model.3.fit)
model.fit.results[4,2] <- AIC(model.4.fit)
model.fit.results[5,2] <- AIC(model.5.fit)
model.fit.results[6,2] <- AIC(model.6.fit)

# Difference in AIC score for each model to the 'best' model
for(i in 1:nrow(model.fit.results)){
  model.fit.results[i,3] <- abs(min(model.fit.results$AIC) - model.fit.results[i,2])
}

# Fit measures
model.fit.results[1,4:8] <- fitMeasures(model.1.fit, c("chisq", "df", "pvalue", "cfi", "rmsea"))
model.fit.results[2,4:8] <- fitMeasures(model.2.fit, c("chisq", "df", "pvalue", "cfi", "rmsea"))
model.fit.results[3,4:8] <- fitMeasures(model.3.fit, c("chisq", "df", "pvalue", "cfi", "rmsea"))
model.fit.results[4,4:8] <- fitMeasures(model.4.fit, c("chisq", "df", "pvalue", "cfi", "rmsea"))
model.fit.results[5,4:8] <- fitMeasures(model.5.fit, c("chisq", "df", "pvalue", "cfi", "rmsea"))
model.fit.results[6,4:8] <- fitMeasures(model.6.fit, c("chisq", "df", "pvalue", "cfi", "rmsea"))

# Sort by delta AIC scores
# model.fit.results <- model.fit.results[order(model.fit.results$deltaAIC),]

model.fit.results
```

```
##   Model      AIC  deltaAIC     chisq df    pvalue       cfi      rmsea
## 1     1 1002.189 0.5990632 15.091353 16 0.5179582 1.0000000 0.00000000
## 2     2 1003.035 1.4448553  5.937145 11 0.8775250 1.0000000 0.00000000
## 3     3 1005.052 3.4617305  5.954020 10 0.8191114 1.0000000 0.00000000
## 4     4 1005.152 3.5613110 16.053600 15 0.3784983 0.9651845 0.04417139
## 5     5 1001.590 0.0000000 12.492289 15 0.6414491 1.0000000 0.00000000
## 6     6 1003.237 1.6466637  8.138953 12 0.7741796 1.0000000 0.00000000
```

```r
# Optional: export table in LaTeX format
library(xtable)
xtable(model.fit.results)
```

```
## % latex table generated in R 3.3.2 by xtable 1.8-2 package
## % Fri Mar  3 11:24:20 2017
## \begin{table}[ht]
## \centering
## \begin{tabular}{rrrrrrrrr}
##   \hline
##  & Model & AIC & deltaAIC & chisq & df & pvalue & cfi & rmsea \\
##   \hline
## 1 & 1.00 & 1002.19 & 0.60 & 15.09 & 16.00 & 0.52 & 1.00 & 0.00 \\
```

```
##   2 & 2.00 & 1003.04 & 1.44 & 5.94 & 11.00 & 0.88 & 1.00 & 0.00 \\
##   3 & 3.00 & 1005.05 & 3.46 & 5.95 & 10.00 & 0.82 & 1.00 & 0.00 \\
##   4 & 4.00 & 1005.15 & 3.56 & 16.05 & 15.00 & 0.38 & 0.97 & 0.04 \\
##   5 & 5.00 & 1001.59 & 0.00 & 12.49 & 15.00 & 0.64 & 1.00 & 0.00 \\
##   6 & 6.00 & 1003.24 & 1.65 & 8.14 & 12.00 & 0.77 & 1.00 & 0.00 \\
##    \hline
## \end{tabular}
## \end{table}
```

Parameters of best fitting model

In the previous section model 5 was identified as best explaining the data. Inspect the model estimated to best explain the data, and create a plot of the model

```
# summary(model.5.fit)

standardizedParameters <- standardizedsolution(model.5.fit)

standardizedParameters[standardizedParameters$op == "~", ]
```

```
##                lhs op             rhs est.std    se      z pvalue
## 10          EPPrate  ~ rangeClutchSize  -0.044 0.148 -0.300  0.764
## 11          EPPrate  ~  songComplexity  -0.118 0.126 -0.935  0.350
## 12          EPPrate  ~      testesSize   0.248 0.140  1.769  0.077
## 13          EPPrate  ~        bodySize  -0.198 0.153 -1.288  0.198
## 14          EPPrate  ~  altitude_range  -0.189 0.144 -1.313  0.189
## 15          EPPrate  ~      range_size   0.229 0.136  1.678  0.093
## 16          EPPrate  ~    male_feeding  -0.497 0.127 -3.929  0.000
## 17          EPPrate  ~       longevity  -0.189 0.146 -1.291  0.197
## 18  songComplexity  ~ rangeClutchSize  -0.060 0.166 -0.359  0.720
## 19 rangeClutchSize  ~      testesSize  -0.347 0.137 -2.537  0.011
## 20 rangeClutchSize  ~        bodySize  -0.138 0.147 -0.943  0.345
## 21 rangeClutchSize  ~    male_feeding   0.284 0.142  1.998  0.046
## 22 rangeClutchSize  ~      range_size  -0.005 0.143 -0.035  0.972
## 23      range_size  ~        bodySize   0.006 0.167  0.037  0.970
## 24       longevity  ~        bodySize   0.513 0.123  4.174  0.000
## 25    male_feeding  ~      testesSize  -0.055 0.163 -0.335  0.737
## 26    male_feeding  ~        bodySize  -0.222 0.160 -1.390  0.165
```

```
xtable(standardizedParameters[standardizedParameters$op == "~", ])
```

```
## % latex table generated in R 3.3.2 by xtable 1.8-2 package
## % Fri Mar  3 11:24:20 2017
## \begin{table}[ht]
## \centering
## \begin{tabular}{rlllrrrr}
##   \hline
##  & lhs & op & rhs & est.std & se & z & pvalue \\
##   \hline
## 10 & EPPrate & \~{} & rangeClutchSize & -0.04 & 0.15 & -0.30 & 0.76 \\
##   11 & EPPrate & \~{} & songComplexity & -0.12 & 0.13 & -0.93 & 0.35 \\
##   12 & EPPrate & \~{} & testesSize & 0.25 & 0.14 & 1.77 & 0.08 \\
##   13 & EPPrate & \~{} & bodySize & -0.20 & 0.15 & -1.29 & 0.20 \\
##   14 & EPPrate & \~{} & altitude\_range & -0.19 & 0.14 & -1.31 & 0.19 \\
##   15 & EPPrate & \~{} & range\_size & 0.23 & 0.14 & 1.68 & 0.09 \\
##   16 & EPPrate & \~{} & male\_feeding & -0.50 & 0.13 & -3.93 & 0.00 \\
##   17 & EPPrate & \~{} & longevity & -0.19 & 0.15 & -1.29 & 0.20 \\
##   18 & songComplexity & \~{} & rangeClutchSize & -0.06 & 0.17 & -0.36 & 0.72 \\
##   19 & rangeClutchSize & \~{} & testesSize & -0.35 & 0.14 & -2.54 & 0.01 \\
##   20 & rangeClutchSize & \~{} & bodySize & -0.14 & 0.15 & -0.94 & 0.35 \\
##   21 & rangeClutchSize & \~{} & male\_feeding & 0.28 & 0.14 & 2.00 & 0.05 \\
##   22 & rangeClutchSize & \~{} & range\_size & -0.00 & 0.14 & -0.03 & 0.97 \\
##   23 & range\_size & \~{} & bodySize & 0.01 & 0.17 & 0.04 & 0.97 \\
##   24 & longevity & \~{} & bodySize & 0.51 & 0.12 & 4.17 & 0.00 \\
```

```
##   25 & male\_feeding & \~{} & testesSize & -0.05 & 0.16 & -0.34 & 0.74 \\
##   26 & male\_feeding & \~{} & bodySize & -0.22 & 0.16 & -1.39 & 0.16 \\
##    \hline
## \end{tabular}
## \end{table}
```

```
# The following can be used to plot the results, but it is not too clear
# semPaths(model.5.fit, "std", title = FALSE,residuals=FALSE, nCharNodes = 0)
```

EPP predictions from all models As there is very little difference in the fit of most of the models tested, a table will be made comparing the regressions of all models

```
eppDataReg <- as.data.frame(matrix(NA, ncol=5, nrow=6))
colnames(eppDataReg) <- c("Model", "est.std", "se","z", "pvalue")

eppDataReg[,1] <-seq(1,6,1)

# A utility function to extract EPP regression estimates from each model,
# and to return whether they are estimated to be significant at the 0.05 level
source("getEPPreg.R")

eppRegResults <- getEPPreg(model.1.fit)

eppRegResults <- rbind(eppRegResults, getEPPreg(model.2.fit))
eppRegResults <- rbind(eppRegResults, getEPPreg(model.3.fit))
eppRegResults <- rbind(eppRegResults, getEPPreg(model.4.fit))
eppRegResults <- rbind(eppRegResults, getEPPreg(model.5.fit))
eppRegResults <- rbind(eppRegResults, getEPPreg(model.6.fit))

eppRegResults
```

```
##   bodySize altitude_range testesSize range_size songComplexity
## 1   -0.189         -0.180      0.237      0.219         -0.115
## 2   -0.197         -0.188      0.247      0.228         -0.118
## 3   -0.195         -0.187      0.246      0.227         -0.116
## 4   -0.186         -0.179      0.234      0.216         -0.113
## 5   -0.198         -0.189      0.248      0.229         -0.118
## 6   -0.198         -0.190      0.249      0.230         -0.118
##   rangeClutchSize longevity male_feeding
## 1          -0.042    -0.181      -0.475*
## 2          -0.044    -0.188      -0.496*
## 3          -0.044    -0.187      -0.491*
## 4          -0.043    -0.178      -0.469*
## 5          -0.044    -0.189      -0.497*
## 6          -0.044    -0.189      -0.498*
```

```
# Combine with model fitting results
full.results.table <- cbind(model.fit.results, eppRegResults)

full.results.table <- full.results.table  <-
  full.results.table [order(full.results.table $deltaAIC),]

xtable(full.results.table)
```

```
## % latex table generated in R 3.3.2 by xtable 1.8-2 package
## % Fri Mar  3 11:24:21 2017
## \begin{table}[ht]
## \centering
## \begin{tabular}{rrrrrrrrrrrrrrrrl}
##   \hline
##  & Model & AIC & deltaAIC & chisq & df & pvalue & cfi & rmsea & bodySize & altitude\_range & testesSi
##   \hline
## 5 & 5.00 & 1001.59 & 0.00 & 12.49 & 15.00 & 0.64 & 1.00 & 0.00 & -0.20 & -0.19 & 0.25 & 0.23 & -0.12
##   1 & 1.00 & 1002.19 & 0.60 & 15.09 & 16.00 & 0.52 & 1.00 & 0.00 & -0.19 & -0.18 & 0.24 & 0.22 & -0.
##   2 & 2.00 & 1003.04 & 1.44 & 5.94 & 11.00 & 0.88 & 1.00 & 0.00 & -0.20 & -0.19 & 0.25 & 0.23 & -0.1
```

```
##   6 & 6.00 & 1003.24 & 1.65 & 8.14 & 12.00 & 0.77 & 1.00 & 0.00 & -0.20 & -0.19 & 0.25 & 0.23 & -0.1[illegible]
##   3 & 3.00 & 1005.05 & 3.46 & 5.95 & 10.00 & 0.82 & 1.00 & 0.00 & -0.20 & -0.19 & 0.25 & 0.23 & -0.1[illegible]
##   4 & 4.00 & 1005.15 & 3.56 & 16.05 & 15.00 & 0.38 & 0.97 & 0.04 & -0.19 & -0.18 & 0.23 & 0.22 & -0.[illegible]
##    \hline
## \end{tabular}
## \end{table}
```

```
# A it of manual entering required so exporting table
write.csv(full.results.table, file="full.results.table.csv", row.names=F)
```

Model fitting with PIC data

```
model.1.fit <- lavaan::sem(model1, data=PICdat)
model.2.fit <- lavaan::sem(model2, data=PICdat)
model.3.fit <- lavaan::sem(model3, data=PICdat)
model.4.fit <- lavaan::sem(model4, data=PICdat)
model.5.fit <- lavaan::sem(model5, data=PICdat)
model.6.fit <- lavaan::sem(model6, data=PICdat)


model.fit.results <- as.data.frame(matrix(NA, ncol=8, nrow=6))
colnames(model.fit.results) <- c("Model", "AIC", "deltaAIC", "chisq", "df", "pvalue", "cfi", "rmsea")

# AIC scores
model.fit.results$Model <- seq(1, 6, 1)
model.fit.results[1,2] <- AIC(model.1.fit)
model.fit.results[2,2] <- AIC(model.2.fit)
model.fit.results[3,2] <- AIC(model.3.fit)
model.fit.results[4,2] <- AIC(model.4.fit)
model.fit.results[5,2] <- AIC(model.5.fit)
model.fit.results[6,2] <- AIC(model.6.fit)

# Difference in AIC score for each model to the 'best' model
for(i in 1:nrow(model.fit.results)){
  model.fit.results[i,3] <- abs(min(model.fit.results$AIC) - model.fit.results[i,2])
}

# Fit measures
model.fit.results[1,4:8] <- fitMeasures(model.1.fit, c("chisq", "df", "pvalue", "cfi", "rmsea"))
model.fit.results[2,4:8] <- fitMeasures(model.2.fit, c("chisq", "df", "pvalue", "cfi", "rmsea"))
model.fit.results[3,4:8] <- fitMeasures(model.3.fit, c("chisq", "df", "pvalue", "cfi", "rmsea"))
model.fit.results[4,4:8] <- fitMeasures(model.4.fit, c("chisq", "df", "pvalue", "cfi", "rmsea"))
model.fit.results[5,4:8] <- fitMeasures(model.5.fit, c("chisq", "df", "pvalue", "cfi", "rmsea"))
model.fit.results[6,4:8] <- fitMeasures(model.6.fit, c("chisq", "df", "pvalue", "cfi", "rmsea"))

# Model 6 is the best fitting model

standardizedParameters <- standardizedsolution(model.6.fit)

standardizedParameters[standardizedParameters$op == "~", ]
```

```
##                lhs op              rhs est.std    se      z pvalue
## 10         EPPrate  ~ rangeClutchSize   0.106 0.143  0.737  0.461
## 11         EPPrate  ~  songComplexity  -0.109 0.097 -1.126  0.260
## 12         EPPrate  ~      testesSize   0.283 0.100  2.837  0.005
## 13         EPPrate  ~        bodySize   0.003 0.120  0.024  0.981
## 14         EPPrate  ~  altitude_range  -0.119 0.118 -1.007  0.314
## 15         EPPrate  ~      range_size   0.302 0.151  1.996  0.046
## 16         EPPrate  ~     male_feeding  -0.532 0.129 -4.141  0.000
## 17         EPPrate  ~       longevity  -0.526 0.146 -3.599  0.000
## 18  songComplexity  ~ rangeClutchSize  -0.246 0.172 -1.427  0.154
## 19  songComplexity  ~      range_size   0.321 0.184  1.742  0.081
## 20  songComplexity  ~  altitude_range  -0.371 0.161 -2.305  0.021
```

```
## 21 rangeClutchSize  ~      testesSize  -0.097 0.112 -0.865  0.387
## 22 rangeClutchSize  ~        bodySize  -0.076 0.108 -0.706  0.480
## 23 rangeClutchSize  ~    male_feeding   0.584 0.099  5.929  0.000
## 24 rangeClutchSize  ~      range_size   0.406 0.108  3.771  0.000
## 25      range_size  ~        bodySize   0.435 0.154  2.816  0.005
## 26      range_size  ~       longevity  -0.751 0.124 -6.050  0.000
## 27       longevity  ~        bodySize   0.566 0.115  4.932  0.000
## 28    male_feeding  ~      testesSize  -0.209 0.160 -1.303  0.193
## 29    male_feeding  ~        bodySize  -0.143 0.162 -0.886  0.376
## 30      testesSize  ~      range_size  -0.327 0.166 -1.966  0.049
## 31      testesSize  ~ altitude_range   0.201 0.172  1.171  0.242
```

```
xtable(standardizedParameters[standardizedParameters$op == "~", ])
```

```
## % latex table generated in R 3.3.2 by xtable 1.8-2 package
## % Fri Mar  3 11:24:22 2017
## \begin{table}[ht]
## \centering
## \begin{tabular}{rlllrrrr}
##   \hline
##  & lhs & op & rhs & est.std & se & z & pvalue \\
##   \hline
## 10 & EPPrate & \~{} & rangeClutchSize & 0.11 & 0.14 & 0.74 & 0.46 \\
##   11 & EPPrate & \~{} & songComplexity & -0.11 & 0.10 & -1.13 & 0.26 \\
##   12 & EPPrate & \~{} & testesSize & 0.28 & 0.10 & 2.84 & 0.00 \\
##   13 & EPPrate & \~{} & bodySize & 0.00 & 0.12 & 0.02 & 0.98 \\
##   14 & EPPrate & \~{} & altitude\_range & -0.12 & 0.12 & -1.01 & 0.31 \\
##   15 & EPPrate & \~{} & range\_size & 0.30 & 0.15 & 2.00 & 0.05 \\
##   16 & EPPrate & \~{} & male\_feeding & -0.53 & 0.13 & -4.14 & 0.00 \\
##   17 & EPPrate & \~{} & longevity & -0.53 & 0.15 & -3.60 & 0.00 \\
##   18 & songComplexity & \~{} & rangeClutchSize & -0.25 & 0.17 & -1.43 & 0.15 \\
##   19 & songComplexity & \~{} & range\_size & 0.32 & 0.18 & 1.74 & 0.08 \\
##   20 & songComplexity & \~{} & altitude\_range & -0.37 & 0.16 & -2.31 & 0.02 \\
##   21 & rangeClutchSize & \~{} & testesSize & -0.10 & 0.11 & -0.87 & 0.39 \\
##   22 & rangeClutchSize & \~{} & bodySize & -0.08 & 0.11 & -0.71 & 0.48 \\
##   23 & rangeClutchSize & \~{} & male\_feeding & 0.58 & 0.10 & 5.93 & 0.00 \\
##   24 & rangeClutchSize & \~{} & range\_size & 0.41 & 0.11 & 3.77 & 0.00 \\
##   25 & range\_size & \~{} & bodySize & 0.43 & 0.15 & 2.82 & 0.00 \\
##   26 & range\_size & \~{} & longevity & -0.75 & 0.12 & -6.05 & 0.00 \\
##   27 & longevity & \~{} & bodySize & 0.57 & 0.11 & 4.93 & 0.00 \\
##   28 & male\_feeding & \~{} & testesSize & -0.21 & 0.16 & -1.30 & 0.19 \\
##   29 & male\_feeding & \~{} & bodySize & -0.14 & 0.16 & -0.89 & 0.38 \\
##   30 & testesSize & \~{} & range\_size & -0.33 & 0.17 & -1.97 & 0.05 \\
##   31 & testesSize & \~{} & altitude\_range & 0.20 & 0.17 & 1.17 & 0.24 \\
##    \hline
## \end{tabular}
## \end{table}
```

```
# The following can be used to plot the results, but it is not too clear
# semPaths(model.6.fit, "std", title = FALSE,residuals=FALSE, nCharNodes = 0)

eppDataReg <- as.data.frame(matrix(NA, ncol=5, nrow=6))
colnames(eppDataReg) <- c("Model", "est.std", "se","z", "pvalue")

eppDataReg[,1] <-seq(1,6,1)
```

```r
#
eppRegResults <- getEPPreg(model.1.fit)

eppRegResults <- rbind(eppRegResults, getEPPreg(model.2.fit))
eppRegResults <- rbind(eppRegResults, getEPPreg(model.3.fit))
eppRegResults <- rbind(eppRegResults, getEPPreg(model.4.fit))
eppRegResults <- rbind(eppRegResults, getEPPreg(model.5.fit))
eppRegResults <- rbind(eppRegResults, getEPPreg(model.6.fit))

eppRegResults
```

```
##   bodySize altitude_range testesSize range_size songComplexity
## 1    0.003         -0.118     0.281*      0.28*         -0.108
## 2    0.003         -0.126     0.299*     0.298*         -0.116
## 3    0.003         -0.118     0.281*       0.3*         -0.109
## 4    0.003         -0.115     0.274*     0.273*         -0.110
## 5    0.003         -0.125     0.296*     0.295*         -0.112
## 6    0.003         -0.119     0.283*     0.302*         -0.109
##   rangeClutchSize longevity male_feeding
## 1           0.113   -0.521*      -0.531*
## 2           0.108   -0.554*      -0.564*
## 3           0.105   -0.523*      -0.528*
## 4           0.110   -0.508*      -0.518*
## 5           0.107   -0.549*       -0.56*
## 6           0.106   -0.526*      -0.532*
```

```r
# Combine with model fitting results
full.results.table <- cbind(model.fit.results, eppRegResults)

full.results.table <- full.results.table  <-
  full.results.table [order(full.results.table $deltaAIC),]

xtable(full.results.table)
```

```
## % latex table generated in R 3.3.2 by xtable 1.8-2 package
## % Fri Mar  3 11:24:22 2017
## \begin{table}[ht]
## \centering
## \begin{tabular}{rrrrrrrrrrllrrll}
##   \hline
##  & Model & AIC & deltaAIC & chisq & df & pvalue & cfi & rmsea & bodySize & altitude\_range & testesSi
##   \hline
## 6 & 6.00 & 200.07 & 0.00 & 30.04 & 12.00 & 0.00 & 0.84 & 0.21 & 0.00 & -0.12 & 0.283* & 0.302* & -0.
##   3 & 3.00 & 203.00 & 2.92 & 28.97 & 10.00 & 0.00 & 0.84 & 0.23 & 0.00 & -0.12 & 0.281* & 0.3* & -0.
##   5 & 5.00 & 218.89 & 18.82 & 54.86 & 15.00 & 0.00 & 0.66 & 0.28 & 0.00 & -0.12 & 0.296* & 0.295* & 
##   2 & 2.00 & 221.04 & 20.97 & 49.01 & 11.00 & 0.00 & 0.67 & 0.31 & 0.00 & -0.13 & 0.299* & 0.298* & 
##   4 & 4.00 & 236.23 & 36.16 & 72.20 & 15.00 & 0.00 & 0.51 & 0.33 & 0.00 & -0.12 & 0.274* & 0.273* & 
##   1 & 1.00 & 250.30 & 50.23 & 88.27 & 16.00 & 0.00 & 0.38 & 0.36 & 0.00 & -0.12 & 0.281* & 0.28* & -
##    \hline
## \end{tabular}
## \end{table}
```

```r
# A bit of manual entering required so exporting table
write.csv(full.results.table, file="PIC.results.table.csv", row.names=F)
```

Finally, create a pdf output of all the final models

```
pdf("allModels.pdf")
semPaths(model.1.fit, "std", title = FALSE,residuals=FALSE, nCharNodes = 0)
semPaths(model.2.fit, "std", title = FALSE,residuals=FALSE, nCharNodes = 0)
semPaths(model.3.fit, "std", title = FALSE,residuals=FALSE, nCharNodes = 0)
semPaths(model.4.fit, "std", title = FALSE,residuals=FALSE, nCharNodes = 0)
semPaths(model.5.fit, "std", title = FALSE,residuals=FALSE, nCharNodes = 0)
semPaths(model.6.fit, "std", title = FALSE,residuals=FALSE, nCharNodes = 0)
dev.off()
```

```
## pdf
##   2
```

References

Pitcher, T. E., P. O. Dunn, and L. A. Whittingham. 2005. "Sperm Competition and the Evolution of Testes Size in Birds." *Journal of Evolutionary Biology* 18: 557–67.

Rosseel, Yves. 2012. "lavaan: An R Package for Structural Equation Modeling." *Journal of Statistical Software* 48 (2): 1–36. http://www.jstatsoft.org/v48/i02/.
